# Long-Term Followup of Adolescent and Young Adult Females with Hypergonadotropic Hypogonadism

**DOI:** 10.1155/2012/862892

**Published:** 2011-12-10

**Authors:** Tsimaris Pantelis, Vrachnis Nikolaos, Iliodromiti Zoe, Deligeoroglou Efthymios

**Affiliations:** 2nd Department of Obstetrics and Gynecology, University of Athens Medical School, Aretaieio Hospital, 115 26 Athens, Greece

## Abstract

The condition characterized by elevated gonadotrophins (gonadotropins elevated into the menopausal range), low sex steroids, and menstrual disorders was previously termed Premature Ovarian Failure (POF). However, over the last two years an effort has been made by many authors to have the term Primary Ovarian Insufficiency (POI) exclusively applied. Irrespective of the term, the condition concerns adolescent and young adult women under 40 years who experience cessation of menstruation for more than 3 cycles (whereas these women in the past had a rhythmic menstrual cycle) or amenorrhea for 4–6 months against the background of a previously disturbed menstrual cycle. Determining the cause of POI is difficult, and it is even harder to deal with problems arising from the paucity of estrogen as well as to draw up the plan for long-term monitoring of these patients. This paper presents long-term therapeutic management strategies concerning emotional health, hormone replacement therapy, maintenance of bone health, family planning, other associated disorders as well as possible research options for the future.

## 1. Introduction 

Several different terms have been used to describe what Fuller Albright in 1942 originally named “Primary Ovarian Insufficiency” (POI) [1, 2]. A search by Cooper et al. of PubMed [3] revealed that the following terms have been used to describe POI: gonadal dysgenesis, premature ovarian failure (POF), premature menopause, early menopause, hypergonadotropic hypogonadism, ovarian dysgenesis, primary ovarian failure, hypergonadotropic amenorrhea, primary ovarian insufficiency, climacterium praecox, or menopause praecox. 

Investigation into POI is very complex, and in the overwhelming majority of cases (90%) a clear-cut cause is not found [4]. The most common causes are presented in Table 1, while rare cases where POI is the single manifestation of an multiorgan syndrome have also been reported [5]. POI occurs through two major mechanisms either follicle dysfunction or follicle depletion.

Diagnosis of POI is confirmed when in the investigation of menstrual disorders the following are observed: FSH levels greater than 30–40 mIU/mL (varying between different laboratories), in two measurements in a one-month period, and estradiol levels less than 50 pg/mL (which indicate hypoestrogenism). The most commonly observed menstrual disorder is secondary amenorrhea, while for younger patients primary amenorrhea is also observed [6]. Bleeding patterns may also include oligomenorrhea, polymenorrhea, or dysfunctional uterine bleeding usually prior to final cessation of menstruation. In the case of teenagers, definitive early diagnosis is difficult because these concomitant disorders are very frequent in puberty and less than 10% of these adolescents suffer ultimately from POI [7]. The reason we need to expedite diagnosis is the rapidity of establishment of osteopenia (up to 60% of adolescents at the time of diagnosis) or, rarely, osteoporosis caused by it [8].

As mentioned earlier, POI results from either rapid atresia of primordial oocytes or the impairment of existing follicles. Ultrasonographic identification of follicles >3 mm, the advent of menstruation, and the measurement of plasma oestrogens do not prove normal ovarian function [9]. Measurement of Anti-Mullerian hormone and inhibin B has been used to estimate remaining functional follicles. According to ACOG (American College of Obstetricians and Gynecologists) the measurements of all the above are not necessary for the diagnosis of POI [10]. 

Therapeutic goals of POI are emotional health, hormone replacement therapy, maintenance of bone health, family planning, and concern about associated disorders [4]. In the event of coexistence of other endocrinologic issues, abnormalities of karyotype or iatrogenic causes of POI, there are several additional diagnostic procedures which must take place, but they are beyond the scope of this paper.

## 2. Emotional Health

An important problem in treating these patients is that it is difficult to announce the medical situation to both the teenage girl and her parents. The most common words young patients use to describe how they feel after receiving the diagnosis are “shocked,” “confused,” and “devastated” [11].The family feel themselves confronted by an immense problem that will affect their daughter's capacity for fertilization while also creating in them a feeling of “stigmatization” as well as an acute sense of anxiety and insecurity [12].

When parents have fully understood the problem of their teenage daughter, they can help her emotionally and further provide her with support at different times when the teenage experiences emotional swings. Parents must be aware that talking about POI with their daughter is an ongoing process. Responses should be short and clear. Dialogue must always be encouraged, and emotional feedback has to be obtained periodically. Should the patient's questions seem irrelevant, parents need to understand what the hidden message is. The patient's outlook should be encouraged to be positive, and her emotional state must be externalized. Parents must help their daughter develop a sense of herself and her purpose beyond biological motherhood. Since the patient may become secretive and withdrawn, both the adult adolescent patient and her parents need to seek out resources for support. Patients' parents should share their faith or sources of hope and understanding with the child [13]. All the above-mentioned procedures are long-lasting and have to form the basis for a peaceful environment in which such teenagers will grow up in. 

## 3. Hormone Replacement Therapy (HRT) 

Contrary to the case of menopausal women, teenagers need to treat POI via long-term therapy, which not infrequently may be necessary even before puberty. The combined hormone therapy (oestrogen + progestin) should not be administered before the completion of puberty (full development of secondary sexual characteristics). Previously there were no guidelines on dosage and styles of hormones. However, recently the ACOG published a Committee Opinion (Number 502) on Adolescent Health Care [10]. Suggested doses and available routes in the prepubertal patient are 25-microgram estradiol-17*β* transdermal patch (or 0.3 milligram conjugated estrogen orally or 0.2–0.5 milligram micronized estradiol orally) and 2.5–5 milligrams/day oral medroxyprogesterone acetate for 12–14 days every 30–60 days (or 100 milligrams/day oral micronized progesterone for 12–14 days every 30–60 days).

In the postpubertal patient treatment options can include combined hormone contraception or hormone replacement. Suggested doses and available routes in this group are 100-microgram estradiol-17*β* transdermal patch (or 0.625–1.25 milligrams/day oral conjugated estrogen) and 2 milligrams/day oral micronized progesterone with 10 milligrams oral medroxyprogesterone acetate daily for 12–14 days every 30–60 days (or 200 milligrams oral micronized progesterone daily for12–14 days every 30–60 days).

The dosage and route of administration of HRT is extremely complex because of the chronicity (many years of treatment) and because during that period of time many changes take place at both the physical and psychological level. There are rare cases where the estrogen needs are greater in younger women than in menopausal women because the first category is required to achieve peak bone mass, while the latter simply seeks maintenance of bone health [7]. The HRT for the period before completion of secondary sexual characteristics should be the smallest possible (estrogen alone) in order to achieve maximum height increase. Progestogen compound must be added when breakthrough bleeding occurs, in order to induce regular withdrawal bleeding.

In virtually all cases treatment should be individualized to the patient. It must be borne in mind that oral administration of any estrogen exposes the liver to higher concentrations than any other tissue. In contrast, a transdermal patch provides estrogen as 17*β*-estradiol allowing easy absorption, rapid metabolism, and low bioavailability. Several studies have been conducted comparing use of oral versus transdermal estrogens. Use of oral estrogens leads to suppression of insulin-like growth factor I (IGF-I) concentration, while transdermal estrogen did not have a negative effect on IGF-I [14]. In case-control studies of postmenopausal women, transdermal estradiol has been associated with a lower risk of venous thromboembolism than has oral estrogen [15]. 

Despite the fact that the final decision for route of therapy remains between any individual patient and her clinician, based on current data, transdermal HRT should be the first choice for adolescent patients [16]. On the other hand, there are several regions around the world, such as the Mediterranean countries, where the warm and humid climate does not allow the use of patches. To date, however, few studies have actually examined whether HRT administration reduces the long-term risks of POF and no evidence-based guidelines for management of POF exist [17]. Despite the fact that HRT is still a controversial topic as regards menopausal women, there is no doubt among physicians that all women with POI must replace their missing steroid hormones (both estrogens and progesterone) for the time period until they reach the usual age of menopause [18]. All patients with POI who take HRT must be aware that they have a 5–10% possibility of conceiving [19] and for this reason they must use barrier contraceptive methods such as a condom or intrauterine devices. The HRT should be discontinued in the event of a positive pregnancy test and the woman must be advised by a fetal specialist as concerns her possible new “needs” of estrogen and progesterone, this time for the safer maintenance of the starting pregnancy. Although combined oral contraceptives (COCs) appear to be an acceptable method for adolescents' HRT, they provide more steroid hormone than is needed for physiologic replacement and are therefore not recommended as first-line management [4]. Alternative methods for HRT, such as percutaneous estradiol gel and transvaginal progesterone gel, have been introduced over the last few years and seem to be well tolerated, reducing the incidence of the psychologic side effects (emotional lability, sleep disorders, depression) of estrogen single therapy [20]. 

## 4. Family Planning and Sexual Function

In cases of POI, couples typically have the options of adoption or egg donation. As mentioned above, women with POI may experience sporadic resumption of ovarian function and spontaneous pregnancies can occur in 5–10% of those with idiopathic POI [19, 21]. Currently, there are no known markers that are associated with an increased rate of remission and there are no therapies that have been shown to restore ovarian function and fertility. Evidence suggests that pregnancies might occur if women with POI managed to suppress their high FSH concentrations, either with ethinyloestradiol or with gonadotrophin-releasing hormone analogues, and then followed ovulation induction protocols using low-dose gonadotrophins [22]. 

Recent results of cryopreservation techniques of ovarian tissue have raised hopes of fertility preservation in women who are due to receive chemotherapy for cancer treatment. An alternative to cryopreservation of ovarian tissue is ovarian stimulation, provided that cancer treatment can be delayed [23]. Oocytes or embryos can be cryopreserved after stimulation. Although there may be future developments in fertility treatment, such as in vivo maturation of oocytes from stem cells or primordial follicles, in vitro fertilization using donor oocytes remains the mainstay of treatment for POI women who wish to become pregnant. However, according to several authors, these pregnancies are at increased risk for first trimester hemorrhage, gestational hypertension, and/or preeclampsia, the delivery of small for gestational age (SGA) neonates, and postpartum hemorrhage, although these findings are controversial [24].

Since patients with POI have estrogen deficiency, it is expected that there will be a degree of vaginal dryness. This leads to reduced arousal, less frequent sexual encounters, and increased pain [25]. The above-mentioned factors diminish the sense of sexual well-being of young women with POI and, in the case of adult patients who are embarking upon their first sexual experiences, can lead to complex psychosexual impairment [26]. Adequate dosage of estrogen replacement and occasionally androgen replacement, along with sexual counseling, may be helpful in the management of sexual dysfunction. 

## 5. Maintaining Bone Health 

Estrogens have important anticatabolic and anabolic effects on bone remodeling. Consequently, estrogen deficiency among patients with POI results in an imbalance between osteoclast and osteoblast activity and a progressive loss of bone mineral density (BMD). Popat et al. reported that women with POI have lower bone density compared to regularly menstruating women [27]. The same research team concluded that delay in diagnosis of POI contributes to reduced bone density by delaying proper therapy. There are no reports that suggest increased likelihood of fracture in patients with primary ovarian insufficiency. 

Since HRT has not proven to be completely effective in prevention of osteoporosis, additional preventive measures such as calcium and vitamin D intake and weight-bearing exercises should be taken. Adult and young women with POI should engage in a variety of exercises, such as walking, jogging, and stair climbing, along with resistance exercises since these not only aid bone acquisition but also contribute to improvement of cardiovascular circulation. The North American Menopause Society guidelines for perimenopausal and postmenopausal women recommend an intake of 1200 mg of elemental calcium and at least 800 to 1000 IU of vitamin D3 per day [28]. 

There is uncertainty (i.e., no clear purpose) as regards the frequency of dual-energy X-ray absorptiometry (DEXA) scanning in adolescents with estrogen deficiency. In a recent Committee Opinion (no. 502), the American College of Obstetricians and Gynecologists refers to the proposal made by a number of experts for annual monitoring of bone density during early to midpuberty in order to document peak bone accrual, and then every 2 years through late adolescence. By contrast, other experts claimed that there is no need for an annual DEXA scan, as the implications of a low bone mineral density result in this population are unclear, given the low risk of fracture and the potential for long-term treatment of osteopenia or low bone mass. Up to now, use of bisphosphonates is not recommended in the young population because of uncertain adverse effects and safety profiles, especially in cases of spontaneous pregnancy. 

Crofton et al. recently published a study stating that the type and profile of hormone replacement are critical and can have considerable effects on the bone health of women with POI [29]. The author concluded that physiological sex steroid replacement (transdermal estradiol, 100 *μ*g daily for week 1, 150 *μ*g for weeks 2–4; vaginal progesterone, 200 mg twice daily for weeks 3-4) has better effects on bone mass acquisition and turnover compared with standard HRT (oral ethinyloestradiol 30 *μ*g and 1.5 mg norethisterone daily for weeks 1–3, week 4 “pill-free”). Further research in this area is needed.

## 6. Cardiovascular Disease

In 1978, the Framingham Study established that there is an association between early menopause and increased mortality from cardiovascular disease, with an estimated 1.8 relative risk of mortality from ischemic heart disease in those with menopause under the age of 40 compared with those with menopause at 49–55 [30, 31]. Kalantaridou et al. published results demonstrating that young women with POF have significant vascular endothelial dysfunction. Early onset of endothelial dysfunction associated with sex steroid deficiency may contribute to the increased risk of cardiovascular disease and mortality in these women. Hormone therapy restores endothelial function within 6 months of treatment [32]. Estrogen deficiency has also been correlated with adverse effects on lipid profile (increased triglycerides, reduced high-density lipoprotein cholesterol)[33], reduced insulin sensitivity [34], and the metabolic syndrome [35], all recognized risk factors for the development of cardiovascular disease. 

Adult and young women with POI should be counseled as to tobacco avoidance, daily exercise for obesity prevention, and an appropriate Mediterranean diet in order to achieve optimal cardiovascular health. Blood pressure screening, lipid profile measurements, and cardiac imaging should be performed and monitored by a specialist. HRT results in an increased carotid pulsatility index [36], decreased blood pressure, improved renal function, and lowered activation of the renin-angiotensin system in this age group. In a comparison drawn between physiological sex steroid replacement regimens (transdermal estradiol and vaginal progesterone) and standard (oral ethinylestradiol and norethisterone) therapy for 12 months, the first cited regimens showed better results in the above-mentioned cardiovascular parameters [37]. Because these therapies are long-lasting, the risk of thromboembolic events such as pulmonary embolism or deep vein thrombosis must be extensively and repeatedly examined.

## 7. Conclusions

Every prepubertal girl, teenager or young woman, who is diagnosed with primary ovarian insufficiency should undergo extensive search by a group of specialists in a referral center while, moreover, benefiting from special handling of her case that will comprise affection and understanding. The followup, which of necessity will be long term, needs to take place twice annually. The final decision for mode of therapy will be one taken mutually between the individual patient and her clinician. Health care providers who counsel these patients must possess comprehensive knowledge of female reproductive biology as well as special sensitivity to the emotional needs of these patients and their parents, especially at the time of diagnosis. Further investigation must also be carried out into the extent and severity of POI complications and due consideration given to the taking of all necessary precautionary measures.

## Figures and Tables

**Table 1:** Common and less common causes of POI.

Most common investigating causes of POI	Less common investigating causes of POI
Chromosomal abnormalities (gonadal dysgenesis with or without Turner syndrome)	Part of a multiple endocrinopathy (i) hypoparathyroidism, (ii) hypoadrenalism, (iii) & mucocutaneous candidiasis

Premutation of X-chromosome (FMR1 gene) Fragile X syndrome	Autoimmune diseases such as (i) autoimmune lymphocytic oophoritis, (ii) dry-eye syndrome, (iii) myasthenia gravis, (iv) rheumatoid arthritis, (v) systemic lupus erythematosus

Damage from chemotherapy or radiation therapy	Viral infections Galactosemia

Surgical oophorectomy (surgical extirpation)	Sarcoidosis
