# Erratum to “Long-Term Followup of Adolescent and Young Adult Females with Hypergonadotropic Hypogonadism”

**DOI:** 10.1155/2012/680569

**Published:** 2012-09-18

**Authors:** Pantelis Tsimaris, Nikolaos Vrachnis, Zoe Iliodromiti, Efthymios Deligeoroglou

**Affiliations:** 2nd Department of Obstetrics and Gynecology, University of Athens Medical School, Aretaieio Hospital, 115 26 Athens, Greece

In the original paper, the list of authors' names was ordered incorrectly; the correct order is Pantelis Tsimaris, Nikolaos Vrachnis, Zoe Iliodromiti, and Efthymios Deligeoroglou.

